# Identification of Signatures of Prognosis Prediction for Melanoma Using a Hypoxia Score

**DOI:** 10.3389/fgene.2020.570530

**Published:** 2020-09-29

**Authors:** Yanhong Shou, Lu Yang, Yongsheng Yang, Xiaohua Zhu, Feng Li, Jinhua Xu

**Affiliations:** ^1^Department of Dermatology, Huashan Hospital, Fudan University, Shanghai, China; ^2^Institute of Dermatology, Shanghai, China

**Keywords:** melanoma, hypoxia score, prognosis, gene signature, prediction model

## Abstract

Melanoma is one of the most aggressive cancers. Hypoxic microenvironment affects multiple cellular pathways and contributes to tumor progression. The purpose of the research was to investigate the association between hypoxia and melanoma, and identify the prognostic value of hypoxia-related genes. Based on the GSVA algorithm, gene expression profile collected from The Cancer Genome Atlas (TCGA) was used for calculating the hypoxia score. The Kaplan–Meier plot suggested that a high hypoxia score was correlated with the inferior survival of melanoma patients. Using differential gene expression analysis and WGCNA, a total of 337 overlapping genes associated with hypoxia were determined. Protein-protein interaction network and functional enrichment analysis were conducted, and Lasso Cox regression was performed to establish the prognostic gene signature. Lasso regression showed that seven genes displayed the best features. A novel seven-gene signature (including ABCA12, PTK6, FERMT1, GSDMC, KRT2, CSTA, and SPRR2F) was constructed for prognosis prediction. The ROC curve inferred good performance in both the TCGA cohort and validation cohorts. Therefore, our study determined the prognostic implication of the hypoxia score in melanoma and showed a novel seven-gene signature to predict prognosis, which may provide insights into the prognosis evaluation and clinical decision making.

## Introduction

Melanoma is one of the highly malignant cutaneous neoplasms with a rising incidence around the world (Hallberg and Johansson, 2013; Domingues et al., 2018), characterized by its strong metastasis rate and poor prognosis (Nakamura and Fujisawa, 2018). Although surgery, chemotherapy, immunotherapy, and radiation have been performed to treat malignant melanoma, the efficacy of therapies remains limited (Domingues et al., 2018). Therefore, investigating the underlying biological mechanism and identifying new therapeutic targets are demanded.

Tumor microenvironment (TME) refers to the biological environment where tumors initiate, locate, and progress (Brandner and Haass, 2013; Roma-Rodrigues et al., 2019). The interaction between tumor and its TME influence the survival, migration, and invasion of tumor cells (Whiteside, 2008). Hypoxia is one of the essential features in the TME, which originates from the proliferation of tumor cells and increased oxygen consumption (Manoochehri Khoshinani et al., 2016). Tumor Hypoxia results in the activation of hypoxia-inducible factor (HIF), which mediates the expression of genes regulating metabolic pathways, pH regulation, DNA replication, and protein synthesis (Al Tameemi et al., 2019). Thus, tumor hypoxia contributes to heterogeneous changes, genetic instability, angiogenesis, and resistance to treatments, which has become an adverse prognostic factor of tumor assessment (Walsh et al., 2014; Jing et al., 2019). Many studies have suggested that hypoxia is related to poor prognosis in solid tumors (Winter et al., 2007; Ward et al., 2013). Likewise, hypoxia is a critical molecular program in melanoma, promoting tumor growth, invasion, treatment resistance, and relapse through the stabilization of HIF and the regulation of hypoxia-related responses (Widmer et al., 2013; Qin et al., 2016). In light of the essential role of hypoxia in melanoma, the detection and assessment of tumor hypoxia plays a critical role in clinical practice.

Assessment of the oxygen concentration, report of physiologic processes involving oxygen markers, and evaluation of endogenous molecules expression are considered as three major groups to detect tumor hypoxia status (Walsh et al., 2014). Deeply understanding the gene characteristics to estimate the degree of hypoxia would help the prognostic evaluation and treatment options. Immunohistochemistry (IHC) and plasma protein assays were developed for determining hypoxia (Russell et al., 2009; Khan et al., 2013). Recently, bioinformatics has been utilized to determine broader signatures. Based on the 26-gene hypoxia signature (Eustace et al., 2013), hypoxia status classifier was administrated in head and neck cancer (Brooks et al., 2019), and hypoxia score was implemented in lung adenocarcinoma (Liu Z. et al., 2020). Up till now, the hypoxia score in melanoma has not been investigated in detail.

**FIGURE 1:**
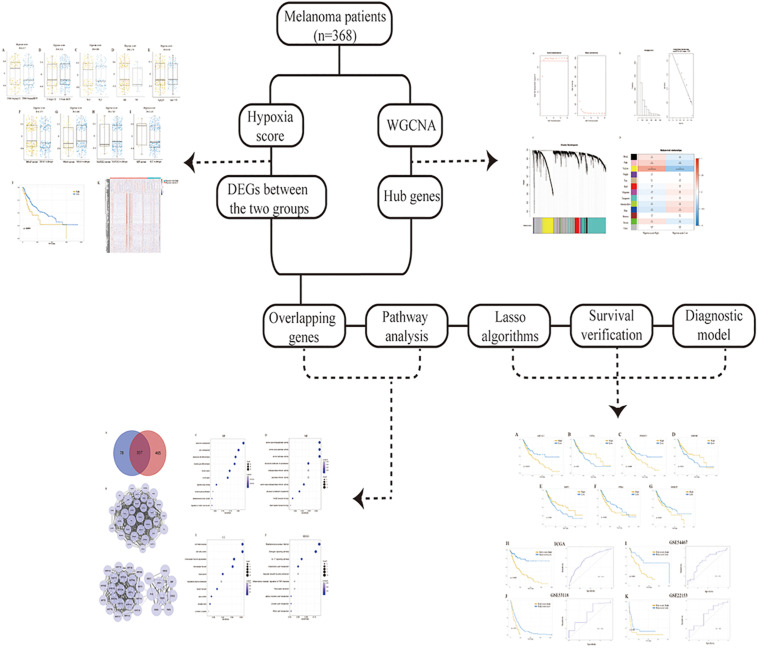
The workflow of the research.

Here, we calculated the hypoxia score for the analysis of gene expression profiles of melanoma which were collected from The Cancer Genome Atlas (TCGA, https://cancergenome.nih.gov). The correlation between hypoxia and prognosis was investigated, and hypoxia-associated molecules were determined. A seven-gene signature was further conducted using the profiles from TCGA and verified in the GSE54467, GSE53118, and GSE22153 dataset, providing novel insights for the assessment, treatment, and prognosis of melanoma. The workflow presenting the design of the present research was shown in Figure 1.

## Materials and Methods

### Data Collection

The clinical information and RNA-sequencing data of skin cutaneous melanoma (SKCM) were downloaded from the TCGA database^1^.

### Calculation of the Hypoxia Score

Hypoxia score was calculated based on the 26-gene hypoxia signature (Eustace et al., 2013) and a gene set variation analysis (GSVA) (Eustace et al., 2013; Hänzelmann et al., 2013). GSVA is a GSE method which estimates variation of pathway activity over a sample population in an unsupervised manner (Hänzelmann et al., 2013). Hence, we used the 26-gene hypoxia signature and evaluated the GSVA score of each sample using the GSVA algorithm. The GSVA score was recognized as the hypoxia score, which represented the hypoxia status of each sample. The cut-off value was identified according to the method of best separation in R package survminer, and patients were divided into high- and low-hypoxia score groups. Such grouping aims to minimize the *P* value of the survival curve. Additionally, *T*-test was used to judge the differences of clinical indexes between groups.

### Definition of Differentially Expressed Genes (DEGs)

EdgeR package was used to identify DEGs between high- and low-hypoxia score groups. The fold change (|fold change| ≥ 1.5) and adj.p < 0.05 were considered significant. Pheatmap package was used to generate the heatmap.

### Identification of Hypoxia-Associated Genes by the Weighted Gene Co-expression Network Analysis (WGCNA)

The top 9829 genes, based on standard deviation, were used for further investigation. Co-expression networks were performed by using the R package WGCNA (Langfelder and Horvath, 2008). Among all the soft threshold values, we chose the β that showed the highest mean connectivity (β = 3). As the module Eigengenes (ME) was recognized to define the interpretation of gene expression profile, we associated the ME with the hypoxia feature, which showed high and low hypoxia score. Module with the highest correlation was selected, and genes of which were named hypoxia-related genes.

### The Protein-Protein Interaction (PPI) Network and Functional Annotation

The overlapping genes between DEGs and hypoxia-related genes were depicted by the online Venn diagram analysis^2^. We used the STRING (version 11.0, Search Tool for the Retrieval of Interacting Genes) and Cytoscape software (version 3.7.0) to construct the PPI network (Shannon et al., 2003; Szklarczyk et al., 2015). Molecular Complex Detection (MCODE) was utilized to determine the interaction clusters. The R package clusterprofile was used to perform functional enrichment analysis and KEGG (Kyoto Encyclopedia of Genes and Genomes) pathway analysis (Yu et al., 2012).

### Survival Analysis and Construction of the Hypoxia-Related Signature for Melanoma

For survival analysis, we utilized Kaplan–Meier survival. Survival-related genes in the multivariate Cox regression analysis were inferred using the least absolutes shrinkage and selection operator (LASSO) by the R package glmnet. Risk scores were obtained according to genes expression multiplied by a linear combination of regression coefficient acquired from the multivariate Cox regression, and patients were divided into a high-risk group and low-risk group based on the optimal cut-off point of risk score using the R package survminer. The Kaplan–Meier analysis and the receiver operating characteristic (ROC) curve were carried out using the R package ROCR.

### Interaction Network Between the 7-Gene Signature and the 26-Gene Hypoxia Signature

To investigate the association between the 7-gene signature and the 26-gene list, genes from these two gene lists were input to the Gene-Cloud of Biotechnology Information (GCBI) analysis platform^3^ for data analysis.

### External Validation of the Hypoxia-Related Signature Model

The signature model was validated using the GSE54467, GSE53118, and GSE22153 dataset derived from the Gene Expression Omnibus (GEO) database^4^. Risk scores were calculated using the same formula, and Kaplan–Meier and ROC curve analyses were implemented.

## Results

### Evaluation of the Degree of Hypoxia

**FIGURE 2:**
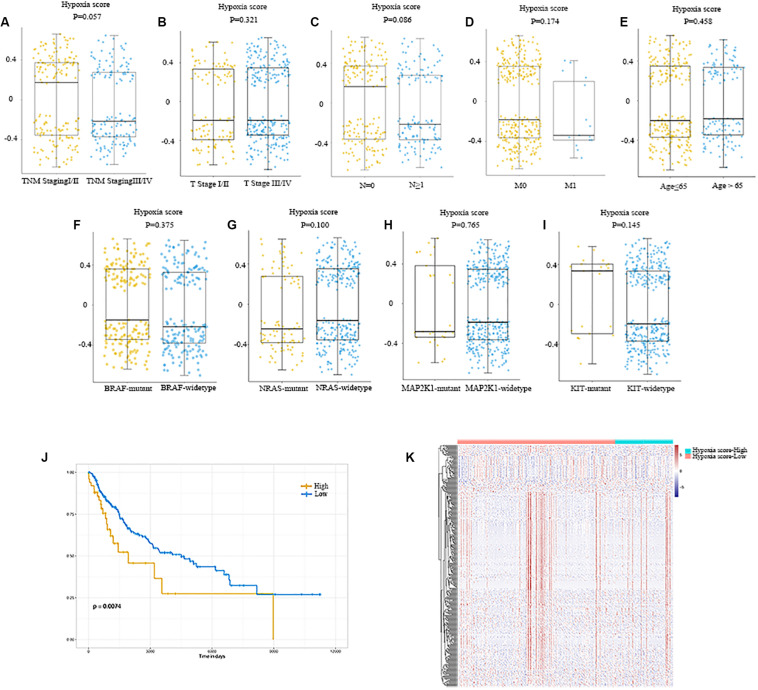
Distribution of hypoxia score in melanoma. **(A)** Distribution of hypoxia score of patients with different TNM staging. **(B)** Distribution of hypoxia score of patients with different T stage. **(C)** Distribution of hypoxia score of patients with or without lymph node metastasis. **(D)** Distribution of hypoxia score of patients with or without distant metastasis. **(E)** Distribution of hypoxia score of patients younger than 65 and those older than 65 years of age. **(F–I)** Distribution of hypoxia score of patients with BRAF mutant and BRAF wildtype, patients with NRAS mutant and NRAS wildtype, patients with MAP2K1 mutant and MAP2K1 wildtype, and patients with KIT mutant and KIT wildtype, respectively. **(J)** Patients were divided into high- and low-hypoxia score groups based on the cut-off value. Patients with a high hypoxia score showed a better prognosis compared to patients with a low score (*(P)* = 0.007). **(K)** Heatmap of the DEGs of high-hypoxia score group vs. low-hypoxia score group. *p* < 0.05, |fold change| ≥ 1.5. DEGs, differentially expressed genes.

Hypoxia scores were distributed between −0.699 to 0.659. A total of 368 patients were divided into high- and low-score groups based on the optional cut-off point of hypoxia score (0.43, Supplementary Figure S1). As shown in Figures 2A–E, no obvious differences in hypoxia scores were detected in patients with different clinical features. Additionally, mutations were common in melanoma, including BRAF (50%), NRAS (30%), MAP2K1 (6%), and KIT (2.6%). So, we also plotted the distribution of hypoxia scores to the status of driver mutations and found they were not significant (*P* = 0.375, *P* = 0.100, *P* = 0.765, *P* = 0.145, Figures 2F–I).

The effects of hypoxia on prognosis were analyzed. The Kaplan–Meier plot suggested that patients with high hypoxia scores had a poor prognosis (*P* = 0.007, Figure 2J). To further determine the correlation of gene expression with hypoxia scores, we did differential gene expression analysis between high and low hypoxia scores. Of the 415 differential expression genes (DEGs), 365 genes were upregulated, while 50 genes were downregulated. Heatmaps in Figure 2K inferred distinct gene expression profiles of cases belong to high- vs. low-hypoxia scores.

### Identification of the Most Relevant Module Genes for Hypoxia in Melanoma

**FIGURE 3:**
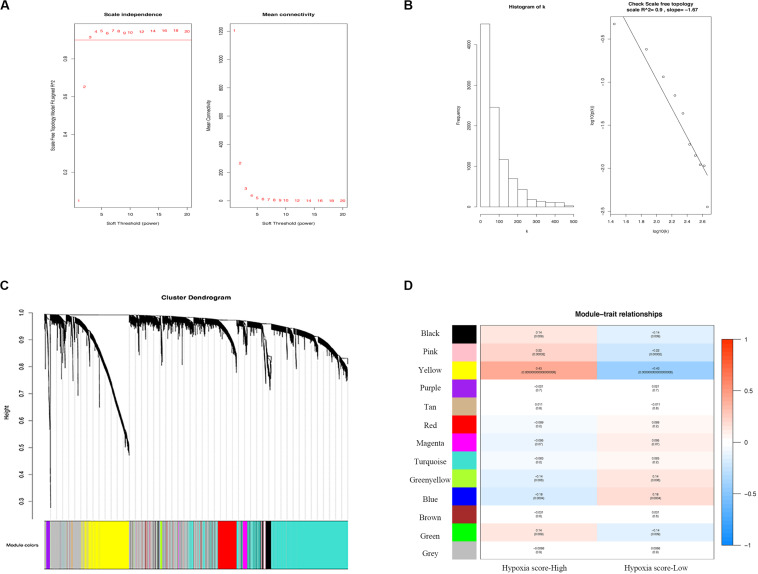
Determination of modules correlated with the hypoxia of melanoma in the WGCNA. **(A)** Analysis of the scale-free fit index and the mean connectivity for various soft-thresholding powers. **(B)** Checking the scale free topology when β = 3. Correlation coefficient = 0.9, which showed scale-free topology. **(C)** Dendrogram of genes clustered according to a dissimilarity measure (1-TOM). **(D)** Heatmap of the correlation between module Eigengenes and hypoxia. WGCNA, the weighted gene co-expression network analysis.

We selected the top 9829 (of 19658) after sorting by the standard deviation (Figures 3A–C). The co-expression network was constructed, and 13 modules were determined. Correlation analysis between the module eigengenes and hypoxia scores showed that the yellow module (Figure 3D, Module–trait relationships = 0.43, *P* = 0.000) had the highest association with the degree of hypoxia. Then, 802 genes in the module were considered to be hub hypoxia-related genes for further investigation.

### Protein-Protein Interactions and Functional Enrichment Analysis

**FIGURE 4:**
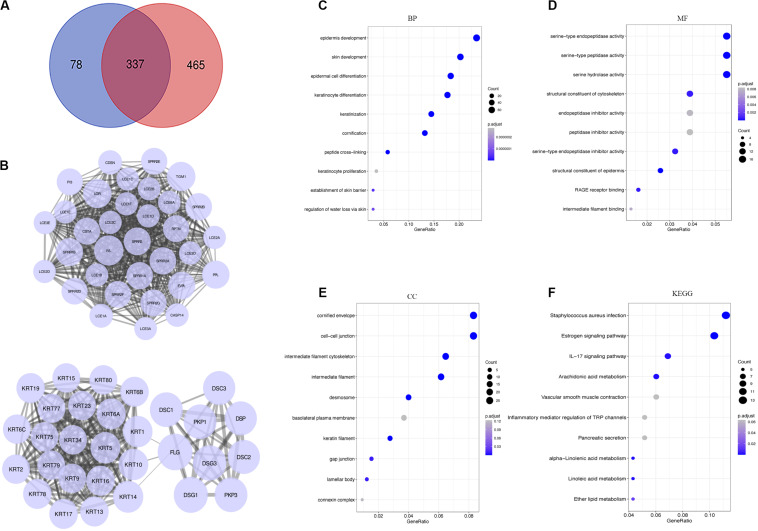
Analysis of DEGs. **(A)** Venn diagrams showing the number of commonly genes in DEGs and yellow module. **(B)** PPI networks of overlapping genes. A large node represented a higher degree. **(C–E)** Go enrichment analysis of biological process (BP), molecular function (MF), and cellular component (CC). **(F)** KEGG pathway enrichment analysis of the overlapping genes. DEGs, differentially expressed genes; PPI, the protein-protein interaction; GO, Gene Ontology; KEGG, Kyoto Encyclopedia of Genes and Genomes.

A total of 337 genes were overlapped between DEGs and hypoxia-related genes (Figure 4A). To explore the interplay among 337 overlapping genes, the STRING tool with confidence > 0.7 was used to construct a PPI network. There were 10 modules in the network, including 195 nodes and 1173 edges. Modules with 10 or more nodes were selected for further analysis (Figure 4B). Based on the connection degree, we named these modules IVL, and FLG modules, respectively. In the IVL module, 528 edges involving 33 nodes were formed in the network. IVL, TGM1, LOR, SPRR1B, and PPL were the remarkable nodes, as they had the most connections with others. In the FLG module, FLG, DSG1, DSG3, PKP3, PKP1, KRT14, and DSC1 occupied the center of the module.

To better understand the biological significance, we conducted enrichment analysis of the 337 overlapping genes. As shown in Figure 4, a total of 27 terms of biological process (BP), 8 terms of cellular component (CC), and 14 terms of molecular function (MF) were enriched (*P* < 0.05). Top GO terms comprised epidermis development, skin development and epidermal cell differentiation (Figure 4C), serine type endopeptidase activity, serine type peptidase activity, and serine hydrolase activity (Figure 4D), and cornified envelope and cell-cell junction (Figure 4E). Besides, KEGG analysis suggested overlapping genes were enriched in *Staphylococcus aureus* infection, estrogen signaling pathway, and IL-17 signaling pathway (Figure 4F).

### Determination of Prognostic Molecules and a Prognostic Risk Model

**FIGURE 5:**
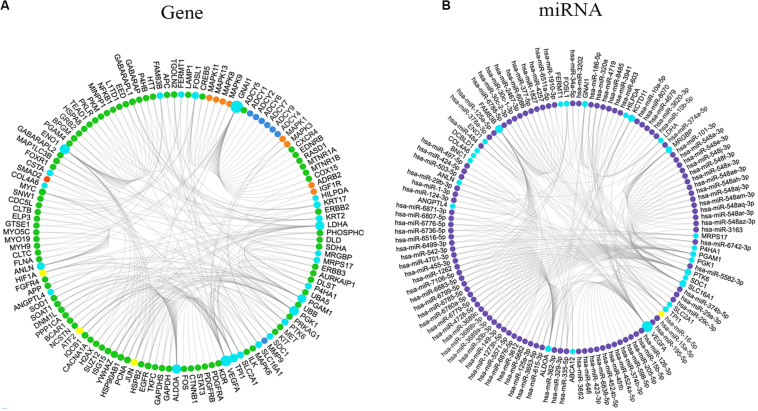
Interaction network of molecules associated with the genes from the 7-gene signature and the 26-gene list. **(A)** Interaction network of genes associated with the 7-gene signature and the 26-gene list. Colors indicated types of genes: light blue, input genes; orange, activated genes; red, expressed genes; green, associated genes; dark blue, inhibited genes; yellow, the largest connection counts. Node size was adjusted according to the number of associated genes. **(B)** Interaction network of miRNAs correlated with the 7-gene signature and the 26-gene list. Colors indicated types of molecules: light blue, input genes; purple, targeted miRNAs; yellow, the largest connection counts. Node size was adjusted according to the number of associated miRNAs.

We generated Kaplan–Meier survival curves to explore the independent prognostic impact of 337 overlapping genes and found that 29 genes were associated with prognosis in the log-rank test (*P* < 0.05). A total of 7 genes identified with the LASSO algorithms included ATP-binding cassette sub-family a member 12 (ABCA12), protein tyrosine kinase 6 (PTK6), fermitin family member 1 (FERMT1), gasdermin C (GSDMC), keratin 2 (KRT2), cystatin A (CSTA), and small proline rich protein 2F (SPRR2F), and constructed as a seven-gene signature model (Table 1). The risk score = 0.26084 * Expression (ABCA12) + 0.05797 * Expression (PTK6) + 0.14404 * Expression (FERMT1) + (−0.44473) * Expression (GSDMC) + (−0.09102) * Expression (KRT2) + (−0.02677) * Expression (CSTA) + 0.11245 * Expression (SPRR2F). The roles of these 7 genes in melanoma and hypoxia responses were described in Table 2. Also, we explored the relationships among genes from the 7-gene signature and 26-gene list. Although genes from the 7-gene signature were different from those of the 26-gene one, there were common regulators associated with hypoxic responses, including EGFR, ERBB2, and miR-125a (Figures 5A,B, Table 3).

**TABLE 1:** The results of Univariate Cox regression analysis.

	HR	Z	P
ABCA12	0.564	−3.171	0.002
PTK6	0.617	−2.687	0.007
FERMT1	0.626	−2.607	0.009
GSDMC	1.547	2.419	0.02
KRT2	1.460	2.105	0.04
CSTA	1.442	2.042	0.04
SPRR2F	0.682	−1.955	0.04

*HR: hazard ratio.*

**TABLE 2:** The roles of 7 genes in melanoma and hypoxia response.

Gene	Function	Role in melanoma	Role in hypoxia response
ABCA12	Membrane transport	Associated with skin malignancies including melanoma	Not reported
PTK6	Protein phosphorylation	Identified as a prognostic biomarker for metastatic skin cancers including malignant melanoma	Up-regulated by HIF-1α and HIF-2α
FERMT1	Keratinocyte proliferation	Not reported	Down-regulated in the condition of hypoxia
GSDMC	Pyroptosis	Present in malignant melanoma and associated with the metastasis	Not reported
KRT2	Keratinization	Not reported	Not reported
CSTA	Keratinocyte differentiation	Not reported	Up-regulated in hypoxic cells
SPRR2F	Epidermis development	Not reported	Not reported

**TABLE 3:** Common regulators and downstream effectors.

	Targeted-genes in the 7-gene signature	Targeted-genes in the 26-gene list
EGFR	PTK6	ALDOA, TPI1
ERBB2	PTK6	KRT17
MiR-125a	FERMT1	VEGFA, ENO1, TPI1

**FIGURE 6:**
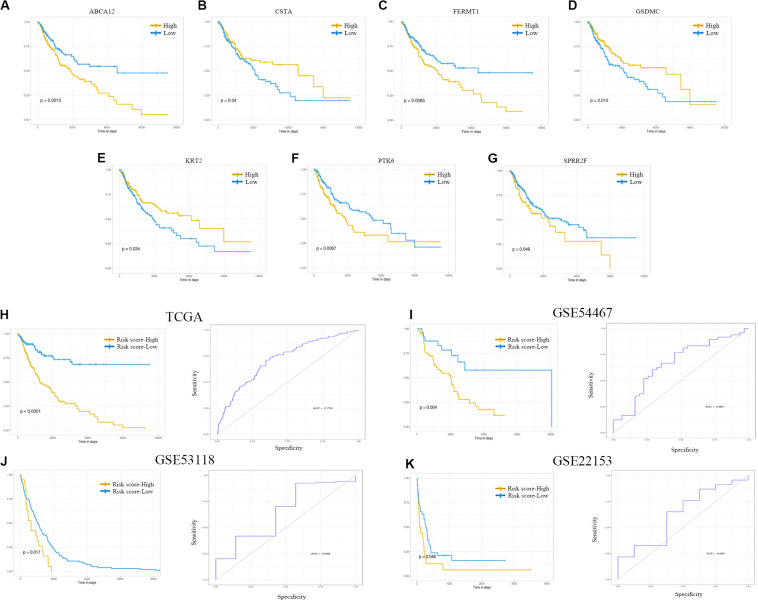
Kaplan–Meier analysis, risk score analysis, and ROC analysis for the seven-gene signature. **(A–G)** Kaplan–Meier curves for overall survival of ABCA12, CSTA, FERMT1, GSDMC, KRT2, PTK6, and SPRR2F. **(H–K)** Kaplan–Meier curves for overall survival of risk score and ROC analysis for the seven-gene signature in TCGA cohort, GSE54467, GSE53118, and GSE22153. ROC, receiver operating characteristic; TCGA, The Cancer Genome Atlas.

Kaplan–Meier curve and ROC were utilized to assess the prognostic capacity of the seven-gene signature model, and similar procedures were performed in the external data. The results showed that genes in the signature model performed well-predicting prognosis within the TCGA cohort (Figures 6A–G). Figures 6H–K suggested that patients with low-risk scores had significantly longer overall survival than those with high-risk scores in TCGA, GSE54467, GSE53118, and GSE22153 dataset (*P* < 0.001, *P* = 0.004, *P* = 0.017, *P* = 0.048). The AUCs were 0.716 (95% CI: 0.661–0.771), 0.667 (95% CI: 0.541–0.792), 0.648 (95% CI: 0.419–0.878), and 0.628 (95% CI: 0.406–0.849), respectively (Figures 6H–K).

## Discussion

Hypoxia, one of the hallmarks of TME, is a biological condition present in most tumors (Jing et al., 2019). Tumor hypoxia exacerbates progression and metastasis through both physiological and genomic mechanisms (Akanji et al., 2019). Investigating crucial features of tumor hypoxia environment may facilitate clinical decision-making.

Previous studies identified several genes, long non-coding RNAs and miRNAs as promising therapeutic biomarkers in melanoma (Zhang et al., 2017; Wei et al., 2019; Xu et al., 2019). However, the differentially expressed signatures were explored between the normal and tumor samples, or between the primary and metastatic tissues, and molecules associated with the progression of cancer were not taken into consideration. Notably, the focus of our study was to estimate the degree of hypoxia according to the evidential basis for 26-gene hypoxia signature (Eustace et al., 2013), and high hypoxia score was demonstrated as a strong predictor of poor clinical outcome. Subsequently, we identified the promising hypoxia-related genes associated with prognosis.

Based on bioinformatics methods and databases, hypoxia score was calculated, and patients were divided into high- and low-score groups. DEGs were collected using differential gene expression analysis. At the same time, WGCNA analysis was performed to select the modules with the strongest relationship between genes in the modules and the module traits. The overlapping 337 genes of the above two clusters were determined as the hypoxia strongly associated genes related to melanoma. Functional analysis showed these 337 genes to be closely related to the development of melanoma, like via cell-cell junction. Cell junction was reported to be relevant for the metastatic process (Knights et al., 2012). Also, the process of epidermis development and epidermal cell differentiation were enriched. Previous studies showed the hyperplastic epidermal region was accompanied by aberrant expression of keratin 14, and melanoma cells were able to increase expression of keratins 8, 19 (Kodet et al., 2015). keratin 8, 14,19 were also observed in the FLG module from PPI network. These results showed that epidermis surrounding melanoma performed hyperplastic features, and indicated the possible interaction between melanoma cells and keratinocytes. KEGG analysis highlighted the estrogen signaling pathway and the IL-17 signaling pathway. Several studies pointed out that the estrogen signaling pathway relied on the balance between estrogen receptor (ER) α and ERβ expression, and the levels of ERβ regulated the capacity of melanoma invasion (Marzagalli et al., 2016; Rajabi et al., 2017). Additionally, IL-17/IL-17RA pathway stimulated cell proliferation of mouse B16F10 and human A375 and A2058 cell lines (Chen et al., 2019). IL-17 and IL-23 immunohistochemistry expression were increased in the melanoma tissues, possibly enhancing VEGF expression and angiogenesis (Ganzetti et al., 2015). Therefore, these analyses supported the hypothesis of the importance of hypoxia microenvironment in the regulation of the biological behavior of tumor cells and surrounding non-tumor cells.

Based on the log-rank test identifying the genes associated with prognosis, LASSO was performed, and seven characteristic variables were extracted. ABCA12 was upregulated in ovarian carcinoma and colorectal cancer, which was recognized as a promising candidate marker (Hlavata et al., 2012; Elsnerova et al., 2016). Mutations in ABCA12 were related to malignant melanoma (Natsuga et al., 2007). PTK6, a non-receptor type tyrosine kinase, was involved in breast, pancreatic cancer and metastatic skin cancer. It was recognized that PTK6 regulated proliferation and migration (Gotoh et al., 2014; Ito et al., 2017; Liu G. et al., 2020). FERMT1, encoding Kindlin-1, was correlated with metastasis and poor prognosis in several solid tumors (Liu et al., 2016; Sarvi et al., 2018). GSDMC functioned as an oncogene, enhancing cell proliferation and tumorigenesis in lung adenocarcinoma and colorectal carcinogenesis (Miguchi et al., 2016; Wei et al., 2020). It presented high in malignant melanoma but undetectable in normal epithelial cells, which might be associated with the metastasis of cells (Xia et al., 2019). CSTA, one of the tumor suppressors, had the anti-apoptotic effect and maintaining cell-cell adhesion. It was upregulated in several epithelial-derived malignancies, including squamous cell carcinoma (Gupta et al., 2015; Ma et al., 2018). KRT2 was found to form a mechanically resilient cytoskeleton and contribute to the skin homeostasis (Fischer et al., 2016). SPRR2F, a cross-linked envelope protein of keratinocytes, providing the protective barrier function (Cabral et al., 2001). Although there was no report of KRT2 and SPRR2F as a prognostic molecule of tumors, KRT2 and SPRR2F might function as promising biomarkers in melanoma. The consistency of our findings regarding ABCA12, PTK6, FERMT1, GSDMC and CSTA with previous studies suggested our method to be reliable, and thus supported the reliability of these potential prognostic and therapeutic targets to a certain extent.

Previous studies inferred that the expression of PTK6 were up-regulated, and FERMT1 were down-regulated in response to the hypoxia condition (Hlavata et al., 2012; Regan Anderson et al., 2013; Lin and Liu, 2019). PTK6 expression depended on both HIF-1α and HIF-2α, which were reported to have a direct regulation of PTK6 transcription. In the analytic process of investigating the effect of hypoxia on the vhl-deficient cells, HIF-regulated genes were obtained. FERMT1 was one of the 214 downregulated DEGs. Additionally, the increased expression of CSTA was detected in hypoxic A431 cells (Park et al., 2010). Although there was no common gene between the 7- and 26-gene signatures, a total of 3 genes, including epidermal growth factor receptor (EGFR), erb-b2 receptor tyrosine kinase 2 (ERBB2), and miR-125a, were identified as common regulators and effectors in these two gene lists in a context-dependent manner. PTK6 was reported to enhance EGFR signaling by direct phosphorylation of EGFR and inhibition of its degradation (Li et al., 2012), and EGFR might promote the cellular response to hypoxia by increasing HIF-1α expression (Swinson and O’Byrne, 2006). Through the split ubiquitin (Ub)-based membrane yeast two-hybrid assay, EGFR was reported to be physically associated with aldolase (ALDOA) and triosephosphate isomerase 1 (TPI1), respectively (Deribe et al., 2009). However, the potential functions of ALDOA and TPI1 need to be further explored. Furthermore, ERBB2, also known as HER2, was recognized as a regulator of HIF-2α and a driver of hypoxic responses (Jarman et al., 2019). PTK6 was coamplified with ERBB2 to promote cell proliferation (Xiang et al., 2008). Additionally, ERBB2 and keratin 17 (KRT17) were found to locate in the same chromosome region, which might have the following tumor associations (Zhang et al., 2013). Apart from regulating the expression of genes, hypoxia-regulated microRNAs (miRNAs) were identified. MiR-125a was a direct target of HIF-1α and drove the reduction of vascular endothelial growth factor A (VEGFA) (Dai et al., 2015; Pan et al., 2018). Based on the map of human miRNA interactome, enolase 1 (ENO1), FERMT1, and TPI1 were observed in the interaction sites of miR-125a and further examinations were demanded (Helwak et al., 2013).

Saxena and Jolly summarized different extents of hypoxia (Saxena and Jolly, 2019). Under acute hypoxia, HIF-1α levels stayed high to regulate acute response, while HIF-2α levels were stabilized later and played a crucial role during chronic hypoxia. Besides, cyclic hypoxia enhanced the expression of HIF-1α instead of HIF-2α. Several factors implicated in these hypoxia conditions were determined, including HSP-70, HAF, H3, H4, REST, and miR-429. Although genes identified in our study have been reported to function in hypoxic responses, there was no report of them to make a distinction of conditions of hypoxia, and further experimental verification is required.

Considering the accuracy of these prognostic genes, a seven-signature model was established based on the combination of genes. Cases in the low-risk group inferred obviously better survival than patients in the high-risk group. The prognosis predictive performance of the model was relatively good not only in the TCGA melanoma cohort but also in the GSE54467, GSE53118, and GSE22153 cohort. Additionally, we investigated whether the clinical features were correlated with the degree of hypoxia, and the results showed that no apparent differences in hypoxia score were observed. BRAF mutation was found to increase HIF-1α expression and influenced survival in previous studies (Kumar et al., 2007; Zerilli et al., 2010). KIT mutant was reported to require HIF-1α to transform melanocytes into melanoma cells (Monsel et al., 2010). In our cohort, the hypoxia score in the BRAF-mutant or KIT-mutant group was slightly higher than that of the wildtype group, but it was not statistically significant. It could be because of an inevitable limitation, the sample size. There were two other limitations to our study. Firstly, data were collected from TCGA, where the potential for selection bias could not be excluded, but we validated the results in the GEO database and demonstrated the reliability to some extent. Secondly, analysis in our study was descriptive, further research *in vitro* and *in vivo* could enhance our understanding of the critical genes.

In conclusion, we applied the hypoxia score to determine the degree of hypoxia in TME and identified the prognostic role of hypoxia score. Furthermore, using bioinformatics and machine learning methods, we determined the seven-gene prognostic signature as a potential prognostic predictor and therapeutic targets for melanoma.

## Data Availability Statement

The datasets presented in this study can be found in online repositories. The names of the repository/repositories and accession number(s) can be found below: https://www.ncbi.nlm.nih.gov/geo/, GSE53118; https://www.ncbi.nlm.nih.gov/geo/, GSE54467; https://www.ncbi.nlm.nih.gov/geo/, GSE22153.

## Author Contributions

XZ, FL, YY, and JX contributed to the design of this study. YS, LY, and YY contributed to the analysis of this study. YS contributed to drafting the text and preparing the tables and figures. All authors participated in the data collection, critical review, revision of this manuscript, contributed to the article, and approved the submitted version.

## Conflict of Interest

The authors declare that the research was conducted in the absence of any commercial or financial relationships that could be construed as a potential conflict of interest.

## Footnotes

1 https://tcga-data.nci.nih.gov/

2 http://bioinformatics.psb.ugent.be/webtools/Venn/

3 https://www.gcbi.com.cn

4 https://www.ncbi.nlm.nih.gov/geo

## Supplementary Material

Supplementary Figure 1 The process of finding optimal cut-off value to divide the patients into high- and low-hypoxia score groups.
