# Supplementary figures and images for: Identification of Signatures of Prognosis Prediction for Melanoma Using a Hypoxia Score
Source: Front Genet. 2020 Sep 29;11:570530. doi: 10.3389/fgene.2020.570530 (PMC7550673; doi:10.3389/fgene.2020.570530)

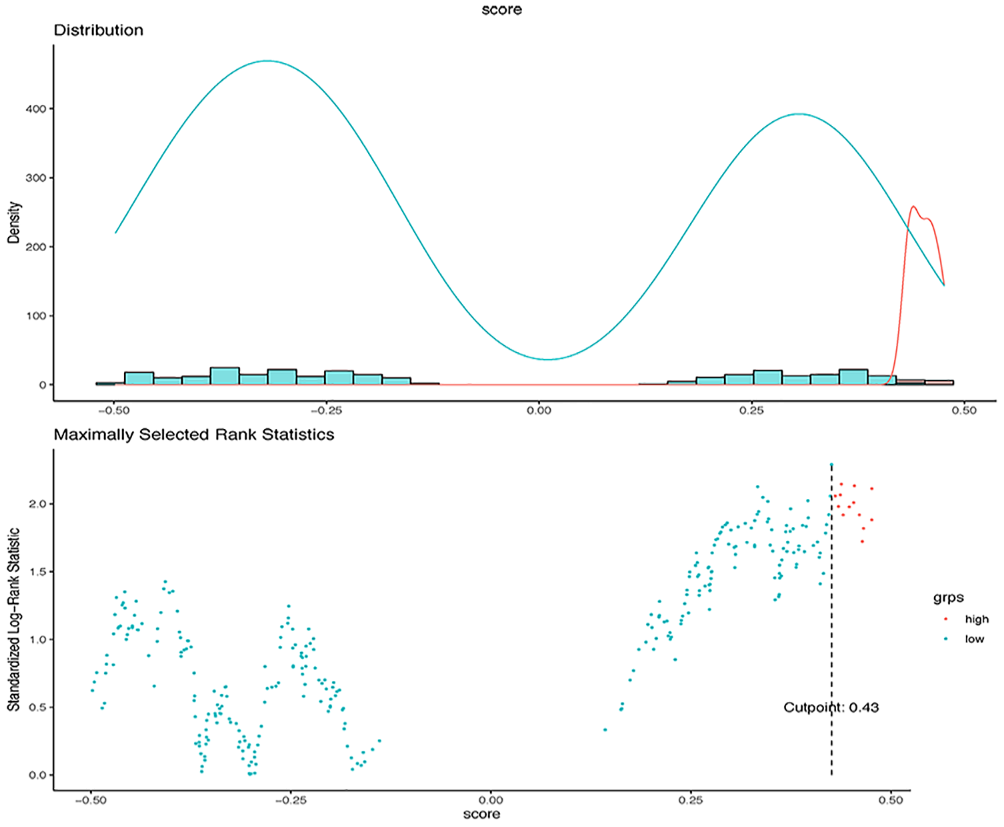

Supplement: Supplementary Figure 1 — The process of finding optimal cut-off value to divide the patients into high- and low-hypoxia score groups. [file Image_1.TIF]
